# The clinical relevance of assessing advanced glycation endproducts accumulation in diabetes

**DOI:** 10.1186/1475-2840-7-29

**Published:** 2008-10-07

**Authors:** Robbert Meerwaldt, Thera Links, Clark Zeebregts, Rene Tio, Jan-Luuk Hillebrands, Andries Smit

**Affiliations:** 1 Department of Surgery, Isala Clinics, Zwolle, The Netherlands; 2 Department of Endocrinology, University Medical Center Groningen, Groningen, The Netherlands; 3 Department of Vascular Surgery, University Medical Center Groningen, Groningen, The Netherlands; 4 Department of Cardiology, University Medical Center Groningen, Groningen, The Netherlands; 5 Department of Cell Biology, University Medical Center Groningen, Groningen, The Netherlands; 6 Department of Vascular Medicine, University Medical Center Groningen, Groningen, The Netherlands

## Abstract

Cardiovascular disease is the major cause of morbidity and mortality associated with diabetes. There is increasing evidence that advanced glycation endproducts (AGEs) play a pivotal role in atherosclerosis, in particular in diabetes. AGE accumulation is a measure of cumulative metabolic and oxidative stress, and may so represent the "metabolic memory". Furthermore, increased AGE accumulation is closely related to the development of cardiovascular complications in diabetes. This review article will focus on the clinical relevance of measuring AGE accumulation in diabetic patients by focusing on AGE formation, AGEs as predictors of long-term complications, and interventions against AGEs.

## Background

Patients with diabetes have a mortality rate from cardiovascular disease (CVD) that is over twice compared to that in the general population [1]. The Adult Treatment Panel III regards diabetes as a coronary heart disease risk equivalent. A number of hemodynamic and metabolic factors co-operate in diabetes [2]. Both the Diabetes Control and Complications Trial (DCCT) in type 1 diabetes mellitus and the UK Prospective Diabetes Study (UKPDS) in type 2 diabetes mellitus established a causal relationship between chronic hyperglycemia and long-term diabetic complications [3,4]. There is increasing evidence that advanced glycation endproducts (AGEs) play a pivotal role in atherosclerosis, in particular in diabetes. AGE accumulation is not just a measure of hyperglycemia, but represents cumulative metabolic burden (both hyperglycemia and hyperlipidemia), oxidative stress and inflammation [5]. Interaction between AGEs and AGE-specific receptors induce inflammatory reactions and endothelial dysfunction [6]. This review will focus on the clinical merits of assessing AGE accumulation in diabetic patients, outlining the evidence for the role of AGEs in the pathogenesis of CVD and the possibilities for AGE-intervention. Finally, we will discuss the clinical relevance for assessing AGE accumulation.

## AGE formation

**Figure 1:**
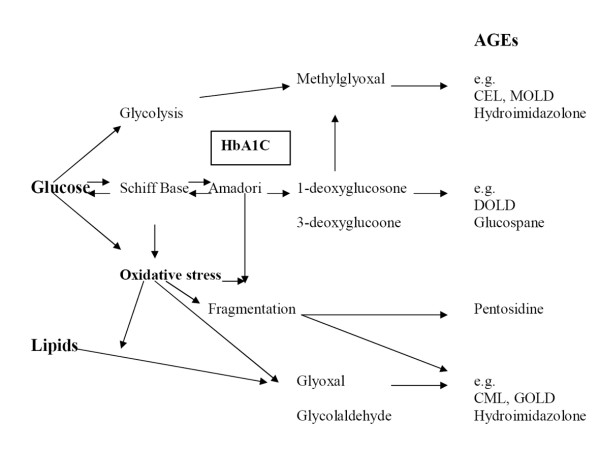
**Simplified scheme of the complex Maillard reaction and formation of some advanced glycation endproducts (AGEs) in vivo**. CEL = carboxyethyllysine; MOLD = methylglyoxal lysine dimer; DOLD, 3-deoxyglucosone lysine dimer; CML, carboxymethyllysine; GOLD, glyoxal lysine dimer.

The original Maillard hypothesis on the formation of AGEs proposed that chemical modification of proteins by reducing sugars (glycation of proteins) in diabetes alters the structure and function of tissue proteins, precipitating the development of diabetic complications (Fig. 1) [7]. Glycation involves the formation of chemically reversible early glycosylation products with proteins, so called Schiff bases and Amadori adducts (e.g. glycated hemoglobin; HbA1C). With time, it became clear that these early adducts undergo slow and complex rearrangements to form advanced glycation end-products (AGEs). Baynes and colleagues noted the importance of oxidizing conditions and reactive oxygen species in the formation of glycoxidation products, the major class of AGEs that accumulate in tissues in diabetes [5]. Besides the formation of carbohydrate intermediates, there is increasing evidence that Maillard products are also formed via lipid-derived intermediates, resulting in advanced lipoxidation products (ALEs)[8]. Dyslipidemia is a common phenomenon in diabetes and lipids are an important source of protein modifications. So, in diabetic patients both AGEs and ALEs may be formed at the same time in atherosclerotic plaques.

Other pathways which may lead to AGE formation is through autoxidation of glucose by reactive oxygen species, and through carbonyl compounds [9,10]. In particular methylglyoxal, a reactive dicarbonyl metabolite of glucose, has received considerable attention as the most reactive AGE precursor in endothelial cells. Decreased clearance of serum AGEs may further increase tissue AGE accumulation and *de novo *formation, and absorption of AGEs from food or smoking may aggravate AGE accumulation in renal failure [11-13].

## Assessment of AGE accumulation

The characteristic fluorescence spectrum of AGEs at 440 nm upon excitation at 370 nm has classically been used to determine tissue AGE accumulation [14]. Later biochemical and immunochemical assays measure both fluorescent AGEs, like pentosidine, and non-fluorescent AGEs, like carboxymethyl-lysine (CML) [15,16]. Complexity, cost and lack of reproducibility contributed to limiting broader use of these latter assays. In recent years, tandem mass spectrometry has considerably facilitated the use and improved the reproducibility of the assay for several AGEs.

Moreover, blood and urine sampling of AGE do not necessarily reflect tissue AGE levels [17,18]. After the formation of AGEs, the accumulation of AGEs bound to proteins is dependent on the half-life of these proteins. On long-lived proteins like skin collagen, lens crystallins and in cartilage proteins, they even accumulate over the lifetime of organisms. Importantly, the sites where chronic complications develop in diabetes are also those where long-lived proteins are present (e.g. glomerular basement membrane, lens crystalline). It seems therefore appropriate to prefer assays of tissue AGE accumulation rather than e.g. plasma samples.

Noninvasive techniques to analyze tissue AGE accumulation, such as lens or skin autofluorescence have also been described. For instance, lens autofluorescence (excitation 350–370 nm, emission 430–450) is significantly higher for diabetic patients than for age-matched control subjects, and the lens autofluorescence increases significantly with the progression of diabetic retinopathy [19]. Several years ago, we developed an instrument, the AGE-reader, designed to noninvasively measure skin autofluorescence. Several studies have shown that skin autofluorescence measured with the AGE-reader is strongly related to AGE accumulation in healthy subjects, diabetic patients and hemodialysis patients [20-22]. Also others have shown an increased skin autofluorescence in relation to AGE accumulation and the screening for type 2 diabetes [23].

## AGE accumulation and CVD

Diabetic patients have a clearly increased risk of cardiovascular morbidity and mortality. Both the DCCT and the UKPDS have shown that hyperglycemia contributes to the increased cardiovascular risk [3,4]. AGEs play an important role in the development and progression of cardiovascular disease in diabetes. Serum levels of AGEs in patients with type 2 diabetes with coronary heart disease (CHD) are increased compared to patients without CHD, and correlate with CHD severity [24]. Even after correction for other cardiovascular risk factors, remain serum AGE levels associated with CHD. AGE deposits have been demonstrated in atherosclerotic plaques and within myocardium fibers [25,26]. Serum levels of AGEs in type 1 diabetic patients are associated with isovolumetric relaxation time of the left ventricle, as a marker of left ventricular diastolic dysfunction [27].

AGE levels are further related to other features of CVD, such as carotid stenosis and peripheral artery occlusive disease. AGE levels are higher in type 2 diabetic patients with peripheral artery occlusive disease compared to those without [28]. Furthermore, AGE contents are correlated to ankle-brachial index, also after correction for other cardiovascular risk factors. The EURODIAB prospective complication study showed a strong correlation between pulse pressure and plasma levels of AGEs in type 1 diabetic patients [29]. Endogenous secretory receptor for AGEs (esRAGE) binds to AGEs and is capable of neutralizing AGE action. In type 1 diabetic patients, circulating esRAGE is inversely correlated with carotid IMT [30]. Koyama et al also observed an inverse relationship between esRAGE and carotid IMT in type 2 diabetic patients and non-diabetic subjects [31]. In humans, RAGE overexpression has been associated with enhanced inflammatory reactions at the vulnerable region of the plaque in carotid endarterectomy specimen [32] In an intervention study it has been shown that statin treatment prior to carotid endarterectomy reduces inflammation as well as RAGE expression [33].

AGEs are not only related to manifestations of CVD, but they also provide prognostic information. Table 1 describes the studies that observed a strong relationship between AGE levels and the development of CVD. Simm et al showed that AGE content correlates with poor outcome as shown by adverse cardiac events in patients after cardiac surgery [34]. AGEs show an inverse relationship with left ventricular ejection fraction, and furthermore correlate with prolonged ventilation time and prolonged stay at the Intensive Care Unit. AGE levels may not only predict operative outcome, but also the success rate of interventions. In diabetic patients receiving cardiac stents an elevated level of serum AGEs appeared to be an independent risk factor for the development of angiographic re-stenosis [35].

**Table 1:** AGEs and the prediction of cardiovascular disease

**Author**	**Year**	**n**	**FU (yr)**	**Tissue**	**Complication**
Simm [34]	2007	75	-	Pericardial	Perioperative cardiac events
Koyama [71]	2007	141	1,5	Serum	Cardiac events (heart failure)
Hartog [38]	2007	102	1,7	Serum	Cardiac events (heart failure)
Kilhovd [37]	2007	386	18	Serum	Cardiac mortality
Meerwaldt [22]	2005	109	3	Skin	Cardiovascular mortality
Meerwaldt [36]	2007	117	5	Skin	Cardiovascular mortality

n = number of patients, FU = follow-up in years, Tissue = tissue in which AGE content was measured.

During long-term follow-up we observed that skin autofluorescence is a strong predictor of survival in diabetic patients [22,36]. Increased serum levels of AGEs predict total, cardiovascular and coronary mortality in women with type 2 diabetes during a follow-up period of 18 years [37]. AGE level remained a strong predictor of survival, even after adjustment for confounding factors, including C-reactive protein. We and others have demonstrated that serum AGE levels are predictors for heart failure and new cardiac events [38,39].

**Figure 2:**
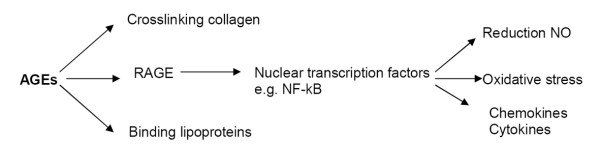
**Pathogenetic effects of advanced glycation endproducts (AGEs).** By binding and crosslinking extracellular matrix, e.g. collagen, AGEs induces vascular stiffness en increases vascular permeability. The interaction with AGE receptors (e.g. RAGE) induces endothelial dysfunction by reducing nitric oxide (NO), inflammatory reactions, and oxidative stress. Binding to lipoproteins increases the uptake of e.g. low density lipoproteins (LDL) by macrophages, which may lead to the formation of foam cells.

Figure 2 shows the mechanisms by which AGEs may contribute to long-term complications. AGEs may especially promote CVD in diabetes through endothelial dysfunction, inflammation, and inducing lipid abnormalities. AGE accumulation may be related to endothelial dysfunction, and endothelial RAGE has been proposed as the major key in such an interaction. Binding of AGEs to RAGE activates endothelial cells, resulting in higher levels of endothelial adhesion molecules like VCAM-1, and activation of transcription factor NF-kB [40]. Endothelial adhesion molecules and NF-kB further increase monocyte adhesivity and vascular permeability, accelerating atherosclerosis [41]. AGEs initiate inflammation-mediated proliferative processes and propagate inflammation in established macrovascular disease [42-44]. Through the interaction with RAGE, AGEs induce oxidative stress. AGEs also induce inflammation by modifying low density lipoproteins, limiting their clearance, promoting uptake by macrophages [45,46].

## AGE accumulation and microvascular complications

Monnier et al first described the relation between AGE accumulation in skin collagen and the severity of long-term diabetic complications [14]. Beisswenger and others have shown that AGE accumulation precedes and correlates with early manifestations of renal disease [47]. Genuth et al were the first to show in type 1 diabetic patients that skin collagen AGE levels (especially carboxymethyllysine) are strong predictor for the development and progression of microvascular complications including nephropathy [48]. Interestingly, the effects of skin AGEs were independent of the preceding HbA1c or those later present during progression of these complications. We recently showed that skin autofluorescence, even after correction for other risk factors, is a strong and independent predictor for the development of microvascular complications, including nephropathy in type 2 diabetic patients [49]. AGEs have a predictive value for the development of microvascular complications, which was found to be superior to other common risk predictors, such as diabetes duration and HbA1c. These results were applicable for primary care type 2 diabetic patients, treated according to current standards. Interestingly, tissue AGE levels prove superior to (single) HbA1c measurements in predicting the progression of diabetic complication [50], as AGE levels may provide a measure of tissue damage resulting from overall metabolic burden, instead of specific risk factors per se [51]. AGE levels in skin collagen predict the risk of future progression of diabetic complications, independently of HbA1c.

Recently, we observed that AGE levels are related to both peripheral and autonomic neuropathy, even before clinical manifestations of neuropathy [52]. Furthermore, skin AGE accumulation is strongly related to the severity of neuropathic foot ulceration. Microvascular disease may worsen ulceration, and endothelial dysfunction has been demonstrated in relation to both diabetic neuropathy and foot ulceration [53]. AGE accumulation has also been reported to worsen endothelial function, and endothelial RAGE has been proposed as the major key in such interaction. Blockage of RAGE accelerates wound closure in diabetic mice and suppresses levels of cytokines such as tumor necrosis factor [54].

## AGE-intervention

Interventions possible against AGE formation and AGE-mediated damage are numerous. Intensive glycemic control in patients from the DCCT trial led to a lower risk for diabetic microvascular complications compared to conventional treatment [3]. The lower risk was associated with lower AGE levels, even after adjustment for HbA1c [3,55]. The discovery of chemical agents that can inhibit glycation reactions may have potential great therapeutic importance. For instance, pyridoxamine, has been shown to inhibit AGE formation and the formation of lipid-derived Maillard products; advanced lipoxidation endproducts (ALEs) [56]. Pyridoxamine is one of the natural forms of vitamin B. It does not directly interact with Amadori products, but interferes with the post-Amadori oxidative reactions. Furthermore, pyridoxamine traps reactive carbonyl compounds, inhibiting AGE and ALE adducts. Pyridoxamine inhibits the development of renal and vascular complications in obese rats [57]. Clinical trials evaluating the efficacy of pyridoxamine in inhibiting the progression of proteinuria and hyperlipidemia in diabetic patients are ongoing. Benfotiamine is a lipophilic derivate of vitamin B1. Its proposed mechanism of action is shunting glycolytic intermediates to the reductive pentose pathway. Benfotiamine reduces the effect of an AGE-rich diet on endothelial dysfunction in type 2 diabetic patients [58].

Another approach has been focused on the cleavage of already formed AGEs protein-protein crosslinks. Although AGE breakers have shown to break AGE-protein crosslinks in vitro, their beneficial effects in trials may not necessarily or exclusively be related to this breaking effect. Compounds like 4,5-dimethyl-3 phenacylthiazolium, also known as ALT-711, have been widely tested. AGE-breakers have been shown to break preformed AGE crosslinks, and to improve arterial compliance in a phase 2 clinical trial in elderly [59]. Currently, clinical trials in heart failure are ongoing (e.g. BENEFICIAL study).

While there are several pharmacological agents specifically aiming at AGEs, there are also "more common" agents that may also reduce AGE accumulation. Interestingly, antihypertensives, such as angiotensin converting enzyme (ACE) inhibitors and angiotensin receptor blockers, reduce AGE accumulation in concert with the severity of diabetic nephropathy [60-62]. Metformine is another examples which has additional effects on AGE accumulation, e.g. by reducing oxidative stress [62-65]. Guanidine compounds block dicarbonyl groups, and metformine (diamino biguanide compound) may decrease AGE accumulation by reducing methylglyoxal levels [66]. Furthermore, metformine is a scavenger of reactive oxygen species [67]. Metformine treatment improves endothelial function in type 2 diabetes and this effect is independent of the antihyperglycemic properties of metformine [68].

Importantly, in the past, clinical evaluation of AGE inhibition has been limited due to concern about long-term toxicity. An important aim of ongoing trials is to monitor unexpected side effects. Without important information on safety or clinical efficacy, trials have mostly focused on patients with long-term complications instead of analyzing primary prevention in diabetic patients.

## AGEs and clinical relevance in diabetes

Table 2 and 3 summarize the potential clinical relevance and a future perspective of assessing AGE accumulation in diabetes. AGE accumulation is a strong predictor for the development and progression of long-term complications, as described above. Many studies observed that AGE levels were related to these events independent of HbA1c, or even found no contribution of HbA1c as a predictor for complications. This might in part be explained by the concept that tissue AGE accumulation reflects cumulative metabolic stress (i.e. metabolic memory) rather than short term glycemic control (HbA1c). AGE accumulation is not just a measure of metabolic stress, but also incorporates oxidative and carbonyl stress. In other words, it shows protein tissue damage resulting from many risk factors for cardiovascular disease.

**Table 2:** Clinical relevance of advanced glycation endproducts (AGEs)

Measure of long-term cumulative metabolic stress
AGEs as a mechanism for the "metabolic memory" observed in diabetes
Measure of oxidative stress (e.g. from smoking) and its' interaction with metabolic stress
Shows the resulting protein damage from various cardiovascular risk factors
AGEs are independent predictors of cardiovascular complications and mortality
Perioperative risk (e.g. cardiac events, pulmonary complications) is related to AGEs
AGEs may help in monitoring and tailoring diabetes treatment

**Table 3:** Future clinical perspective of advanced lycation endproducts

Clinical trials with various anti-AGE interventions
Feasibility studies in daily clinical practise:
-metabolic control (e.g. HbA1c vs AGEs)
-tailoring treatment
-risk analysis of interventions (e.g. surgery)
Role of AGEs in subjects with impaired (fasting) glucose tolerance
Identifying the "vulnerable patient" at risk for cardiovascular disease in e.g. primary care

It is also important to stress that there is a major overlap in (increased) cardiovascular risk between persons with impaired (fasting) glucose tolerance and type 2 diabetes. The diagnosis of diabetes is also not a fixed diagnosis over time in e.g. an obese patient who manages to reduce weight considerably. If metabolic imbalances are stronger determining factors for cardiovascular risk than momentary glucose levels, or than a diagnosis of diabetes, this supports a role for assessing AGE accumulation.

Measuring tissue AGE levels may be useful in monitoring and tailoring diabetes treatment. Many patients already use for example statins, ACE inhibitors, or metformine, which all may reduce AGE accumulation. Unfortunately, clinical trials evaluating the effect of specific AGE inhibitors or breakers are limited or still in progress. Some early trials were hampered by side effects of these pharmacological agents (e.g. vitamin B6 deficiency syndrome) [69].

The question remains whether AGEs are a risk factor for CVD in the long run, or that AGEs are also a measure of current CVD activity. If AGE levels help to identify the diabetic patient with unstable cardiovascular disease, this may definitely increase the clinical relevance. Epidemiological studies investigating AGE levels as a marker of vulnerable plaques are lacking. Nevertheless, we recently observed that skin autofluorescence is an independent marker of acute myocardial infarction and it predicts major adverse cardiac events one year after acute myocardial infarction (personal data: Mulder et al., American Heart Association 2006). Interestingly, skin autofluorescence was strongly related to markers of inflammation and oxidative stress.

Finally, it is important for clinicians to critically appraise the value of new cardiovascular risk factors. As described by Morrow et al., the value of new risk factors may be structured around three issues: can we measure the risk factor, does it add new information and does it help to manage patients [70]? AGEs can be measured as described above, although limitations remain. AGE levels add new information for the development and progression of cardiovascular disease in both diabetic and non-diabetic patients. Studies to test whether AGE levels really help to manage diabetic patients in daily practice are now in progress.

## Conclusion

AGEs have been implicated in the pathogenesis of long-term complications in diabetes. Animal and *in vitro *research has shown that AGEs affect extracellular proteins and activate cytokine production and transcription factors via binding to AGE receptors. AGE accumulation closely correlates with the severity and predicts the development of cardiovascular complications. A variety of interventions against AGE accumulation, predominantly tested in preclinical contexts, appear to show beneficial effects on the development/progression of diabetic complications. Findings from current clinical trials, including feasibility studies, may further help in determining the relevance of AGE assessment in diabetes.

## Abbreviations

AGEs: advanced glycation endproducts; ALEs: advanced lipoxidation endproducts; RAGE: receptor for advanced glycation endproducts; CVD: cardiovascular disease; CHD: coronary heart disease; ACE: angiotensin converting enzyme; VCAM: vascular cell adhesion molecule; IMT: intima-media thickness; CML: carboxymethyllysine; HbA1c: glycated hemoglobin.

## Competing interests

Dr AJ Smit is co-founder of Diagnoptics, manufacturing the AGE-reader. Final approval and responsibility for the current review is with the first author R Meerwaldt.

## Authors' contributions

RM was responsible for writing the review article and final approval. TL was responsible for the design of the included studies (literature search). CZ was responsible for the literature search and revision review article. RT was responsible for the revision review article. JLH was responsible for the writing section on AGE formation. AS was responsible for drafting the review article.
